# Hard-Flaccid syndrome: a survey of sexual medicine practitioners’ knowledge and experience

**DOI:** 10.1038/s41443-024-00917-3

**Published:** 2024-06-19

**Authors:** Gustavo Gryzinski, Muhammed Moukhtar Hammad, Laith Alzweri, Babak Azad, David Barham, Supanut Lumbiganon, Ege Can Serefoglu, Faysal Yafi

**Affiliations:** 1 University of California, Irvine Department of Urology, Irvine, CA USA; 2 The University of Texas Medical Branch at Galveston Division of Urology, Galveston, TX USA; 3 Biruni University School of Medicine Department of Urology, İstanbul, Türkiye

**Keywords:** Sexual dysfunction, Diagnosis

## Abstract

Hard-flaccid syndrome (HFS) is a poorly understood condition of male sexual dysfunction (MSD) that has more recently become a new topic of discussion in online forums and sexual medicine conferences. There has been limited research looking into HFS and consequently there are no evidence-based guidelines for its work-up and management. In order to identify the current level of understanding of HFS in the sexual medicine community, a survey was distributed at a national urologic conference focusing on pertinent management strategies employed by practitioners, and their own thoughts on HFS. This showed that nearly one-third of those surveyed had never seen HFS in their practice. Of those that had, diagnosis was mainly made via clinical history as well as patient self-diagnosis. Additionally, only about half of the respondents who had seen HFS were confident in its legitimacy as a real medical syndrome. This analysis is one of the first of its kind, and highlights the ongoing lack of familiarity of HFS among the sexual medicine community. There were limitations, most notably its survey format as well as low sample size, however, it importantly emphasizes the critical need for continued education and research into HFS to improve diagnostic accuracy, enhance patient care, and develop effective treatment strategies.

## Introduction

Hard-flaccid syndrome (HFS) is a condition of male sexual dysfunction (MSD) that has become a relatively new focus in online forums and urologic conferences [1, 2]. It is a poorly understood syndrome characterized by chronic pain, a partially rigid penis while in a flaccid state, sensory changes mostly at the glans, and erectile dysfunction [1]. Interestingly, there are also reports of having the pain exacerbated during ejaculation and micturition. Additionally, this painful and psychologically worrisome condition results in significant anxiety and depression with significant impacts on quality of life [1, 3, 4].

HFS primarily affects men in their second and third decade of life, but its exact pathophysiology remains uncertain, with hypotheses most commonly suggesting an initial traumatic injury to the base of the penis typically during sexual intercourse [1]. Specifically, damage to the neurovascular bundles that supply innervation to the penis and pelvic floor. The pathophysiologic process is thought to be an emotional reaction following injury and noticing the sensory changes on the penis which then causes a feedback loop where this reaction triggers a hypersympathetic response leading to extensive pelvic floor muscle spasms which ultimately causes additional extrinsic damage to the penile neurovascular bundles [1, 4]. Work-up including cross-sectional imaging, penile ultrasound doppler, and labs tests tend to be completely normal [1, 5]. Regarding the treatment of HFS, there have been combinations utilized with pelvic physical therapy, lifestyle and behavioral modifications, antidepressants, and phosphodiesterase 5 (PDE5) inhibitors. However, there has not been strong evidence regarding their efficacy. Additionally, there are conflicting results for certain therapies, particularly medical therapy with PDE5 inhibitors [1, 3].

Given the paucity of studies and lack of established guidelines regarding HFS, an evidence-based understanding is lacking within the sexual medicine community, and this as understood as most of the published knowledge regarding HFS has been extracted from online forums and chat groups [1]. In order to better understand this condition and how to manage patients adequately, the current level of understanding of HFS first must be established. Because of this, we sought to query a group of contemporary clinicians on their baseline comprehension of HFS and how they diagnose and manage their patients.

## Materials and methods

A survey was provided to sexual medicine practitioners at the 2023 American Urological Association (AUA) Annual Meeting who attended a plenary focusing on sexual medicine. Each survey form included a screening question to determine familiarity with HFS. If they were familiar with HFS, there were multiple additional questions querying experience with HFS in their patients, modalities of diagnosis, preferred therapy options, in addition to others. The investigators were blinded to the respondents’ answers. These responses were then extracted onto a spreadsheet and analyzed.

## Results

According to the registration reports from the 2023 AUA conference, there were 37 attendees at the sexual medicine plenary. A total of 36 survey responses were submitted. Of these, 35 were urologists and 1 responder was a mental health professional. 23 of the 36 respondents (64%) had seen patients with HFS in their practice. Practitioners who had not seen HFS did not complete the rest of the survey. Among the respondents, 8 (35%) first acquired knowledge about HFS through their colleagues, 6 (26%) from medical journals, 4 (17%) from conferences, 4 (17%) from their patients, and 1 (5%) reported that they first learned about HFS from an online pelvic floor therapist. Regarding the diagnostic approach for HFS in their practice, clinical history was utilized by 17 responders, self-diagnosis by 16 responders, and 1 utilized imaging, although the specific imaging modality was not specified. Most respondents (74%) indicated that they had seen 1-10 patients with HFS, 4 respondents saw between 11–20 patients, and 2 practitioners saw 21–50 patients. 18 of the 23 respondents who had seen patients with HFS reported a history of urogenital trauma during their evaluation. The most common physical exam findings were having a semi-erect penis in the flaccid state (12), followed by contracted perineal muscles (8), coldness in the glans/shaft of the penis (6), trigger points in the perineal muscles (6), and exaggerated bulbocavernosus reflex (3). However, up to 43% of practitioners surveyed found that the physical exam was always normal. Regarding imaging, penile duplex doppler ultrasound was utilized by 8 respondents, 7 of which reported to sometimes have abnormal findings. A variety of treatment modalities were employed including pelvic floor therapy, PDE5 inhibitors, behavioral modifications, mental health specialist referrals, alpha-blockers, shockwave therapy, antispasmodics, antidepressants, and muscle relaxants. The majority of respondents (11) suggested pelvic floor therapy as the optimal approach, with 10 recommending combination therapy which was typically with medical and/or behavioral therapy. Of the 23 respondents, only 13% felt they were successful in their management of their patients’ HFS, 30% felt they were not successful, and the remaining 57% felt they were sometimes successful. In terms of their beliefs about HFS as a legitimate condition, 12 responders expressed confidence in its existence, while 7 remained uncertain, and 4 practitioners did not believe it is a real condition.

## Discussion and conclusion

This study highlights the ongoing lack of familiarity with HFS not only in the urologic field in general, but in the sexual medicine society specifically. There are some notable limitations, namely the survey format and small sample size. Despite these limitations, this article is one of the first of its kind and provides valuable insights into the current understanding of this complex condition. It suggests that a significant portion of sexual medicine practitioners have limited knowledge of HFS, particularly its presentation, work-up, and management. Importantly, even in those who have seen HFS in their practice, a large portion still have doubts regarding its legitimacy as a diagnosis. There is a lack of concrete diagnostic criteria to establish the diagnosis as the vast majority of diagnoses were made based on solely clinical history and patient self-diagnosis, which is similar to what has been described in the literature [1]. Similarly, there was a wide variety of diagnostic modalities utilized. There does seem to be a general consensus that either pelvic floor therapy in isolation or combined with other modalities such as medical and/or behavioral therapy is the optimal treatment approach which has been also described in the literature through the use of biopsychosocial treatment approaches, however with spotty success [4].

Ultimately, these findings emphasize the critical need for continued education and research on HFS in order to improve diagnostic accuracy, enhance patient care, and develop evidence-based, effective treatment strategies as these patients experience a physically and emotionally damaging ailment that very few sexual medicine clinicians are comfortable managing. Future work should be dedicated towards more basic science research into HFS to further enforce its legitimacy and ensure it becomes recognized as a legitimate condition with the ultimate goal of improving diagnostic accuracy via specific criteria, enhance patient care and outcomes, and develop effective treatment approaches with adequate accessibility.
